# A. Cornelius Celsus

**Published:** 1913-07-19

**Authors:** 


### MEMOS. FROM THE MASTERS.

### A. Cornelius Celsus.

To let blood is very easy—to one thereunto accustomed, having experience. Yet to an ignorant person it is very difficult. For the vein is close to the arteries and nerves, and if the lancet touches a nerve convulsions follow and cruelly destroys the patient, while a cut artery neither unites nor heals, and may be the cause of sudden hæmorrhage. If the lancet is introduced timidly it merely lacerates the skin and does not cut the vein. Sometimes also the vein is hidden and not easily recognisable. Thus it is seen that many things render difficult to a novice what is really very simple and easy to an expert. The vein is to be cut in the middle, and when it is opened it is of importance to notice what colour the blood has and what its condition is. Blood that is thick and black is bad, and it is therefore properly discharged, while if it is red and clear it is sound, and the letting of it does not benefit and should be stopped. That indeed cannot happen under a physician, who knows from what body it is beneficial to let blood. When the flow has been judged sufficient it should be stopped; in any case it should be stopped before the patient's mind forsakes him. Then the arm must be bound up with a pellet of wool pressed out in cold water put upon the wound. On the next day the course of the vein is to be rubbed with the opposite middle finger that the recent union may be dissolved and it may pour out blood again. When, however, that blood so let, which before was black and thick, is on this second letting found to be clear and red, the arm is at once to be bound up and to be kept guarded until there is a strong cicatrix. (*De Re Medica. Liber II.*)

### Of Bedside Tact.

Fear and excitement weaken and harass the sick. Therefore when the physician first enters the sickroom the anxiety of the patient, doubting how the case may appear, causes the latter's pulse rate to rise. So it is the physician's duty, if he is skilful, not directly upon entering the room to grab the patient's arm, but to sit down with a cheerful countenance, and to talk pleasantly, encouraging the timid patient by plausible conversation, and finally to feel the pulse. How easily do a thousand things or even the sight of the physician disturb the vessels! (*De Re Medica. Liber III.*)

